# Gold nanoparticles with chitosan, N-acylated chitosan, and chitosan oligosaccharide as DNA carriers

**DOI:** 10.1186/s11671-019-3083-y

**Published:** 2019-07-30

**Authors:** Paulina Abrica-González, José Alberto Zamora-Justo, Antonio Sotelo-López, Guillermo Rocael Vázquez-Martínez, José Abraham Balderas-López, Alejandro Muñoz-Diosdado, Miguel Ibáñez-Hernández

**Affiliations:** 1 Instituto Politécnico Nacional, Basic Sciences Department, Unidad Profesional Interdisciplinaria de Biotecnología, 07340 Mexico City, Mexico; 2 Instituto Politécnico Nacional, Biochemistry Department, Escuela Nacional de Ciencias Biológicas, 11340 Mexico City, Mexico

**Keywords:** Gene therapy, Gold nanoparticles, Chitosan, Transfection

## Abstract

Currently, gold nanoparticles have found applications in engineering and medical sciences, taking advantage from their properties and characteristics. Surface plasmon resonance, for instance, is one of the main features for optical applications and other physical properties, like high density, that represents the key for cellular uptake. Among other applications, in the medical field, some diseases may be treated by using gene therapy, including monogenetic or polygenetic disorders and infections. Gene adding, suppression, or substitution is one of the many options for genetic manipulation. This work explores an alternative non-viral method for gene transfer by using gold nanoparticles functionalized with organic polymers; two routes of synthesis were used: one of them with sodium borohydride as reducing agent and the other one with chitosan oligosaccharide as reducing and stabilizing agent. Gold nanoparticles conjugated with chitosan, acylated chitosan and chitosan oligosaccharide, were used to evaluate transfection efficiency of plasmid DNA into cell culture (HEK-293). Physical and chemical properties of gold nanocomposites were characterized by using UV-Vis Spectroscopy, ξ**-**potential, and transmission electron microscopy. Furthermore, the interaction between gold nanoparticles and plasmid DNA was demonstrated by using agarose gel electrophoresis. Transfection tests were performed and evaluated by β-galactosidase activity and green fluorescence protein expression. The percentage of transfection obtained with chitosan, acylated chitosan, and chitosan oligosaccharide were of 27%, 33%, and 60% respectively.

## Background

**Fig. 1:**
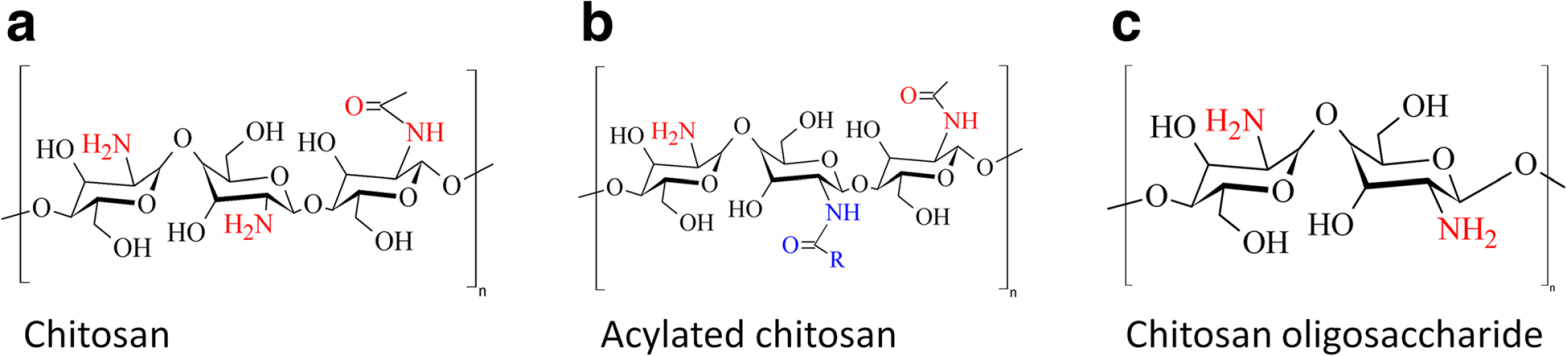
Chemical structure of **a** chitosan, **b** acylated chitosan, and **c** chitosan oligosaccharide

Gene therapy can be briefly defined as a way of introducing genetic material into cells for different purposes, the treatment of genetic diseases among them [1]. Among the two main types of DNA vectors for gene therapy, viral and non-viral, the last one has the advantages of absence of immunogenicity, good compliance, non-infectivity [2], in-storage stability, and ease of production. On the other hand, even when viral vectors such as retroviruses, adeno-viruses, adeno-associated viruses, etc., have high transfection rates and fast transcription, they present drawbacks such as rapid clearance from circulation, toxicity, immunogenicity, and reduced capacity to carry large amounts of information [3–5]. Usually, in the case of non-viral carriers, the foreign DNA is loaded onto a plasmid and it is protected and stabilized throughout the formation of conjugates or complexes. In particular, chitosan (CO) has been studied for the development of non-viral DNA vectors, since it has shown high transfection rates and low toxicity, protecting the DNA from the nucleic acids during intercellular transport [4]. This polymer widely present in nature (it is found as the main constituent of crustaceans shells and as part of the cell walls of many fungi [6]) is a polysaccharide that can be obtained after chitin transformation (obtaining 2-amino-2-deoxy-β-D-glucopyranose repeating units but still retaining a small amount of 2-acetamido-2-deoxy-β-D-glucopyranose residues [7]) (Fig. 1a). Medical applications of native chitosan require, in most cases, some modifications of the polymer; among the main disadvantages to overcome are insolubility in physiological pH and high viscosity in dilute acid solution [8]. Some researchers have pointed out that cell transfection dynamics depends on vehicle’s size and shape [1, 8–12], for instance, Huo et. al. reported how these properties correlate with penetration efficiency for the case of gold nanoparticles [10].

Chitosan solubility can be modified changing the distribution of remaining acetyl groups in the chain and the degree of acetylation [13]. Another method to control the physical properties of chitosan is the acylation (Fig. 1b). According to the availability of amino groups and the grade of acetylation, some bio-interactions of chitosan may be controlled, increasing its hydrophobicity by acylation with fatty acids, for instance [7]. Le Tien et. al. [7] have reported chitosan N-acylation for controlled release matrices in pharmaceuticals. Bhattarai et. al. [14] obtained N-acylated chitosan stabilized gold nanoparticles for applications in physiological conditions, exhibiting the advantages of non-acylated chitosan. Another useful modification of chitosan is the reduction of the polymer chain length (Chitosan oligosaccharide, COS) [15]. Among the properties of the many kinds of COS obtained from chitosan, their lower viscosity and higher solubility in water are very useful for many applications [16]. It has been reported that chitosan of low molecular weight may increase cellular uptake, mainly because of its shorter chain lengths (2-10 D-glucosamine units) (Fig. 1c) and free amino groups [17–19]. In addition, synthesis of gold nanoparticles with chitosan oligosaccharide as reducing agent does not include any toxic reagent [20].

Gold nanoparticles (AuNPs) led to the development of a new kind of nanomaterials, taking advantage of their optical properties and high physical stability that make them a very viable option for several applications [21–25]; hereby, they present improvements for cellular transfection over lipocomplexes or polymers, by having higher density and so reducing uptake time [12]. As gold has a great affinity to amino (-NH_2_), cyano (-CN), and thiol (-SH) functional groups, vast opportunities are open for many types of functionalization [9, 26]. Cationic monolayers are obtained, for instance, by adding cationic polymers to gold nanoparticles, improving nucleic acid interaction and electrostatic adsorption [27, 28]. In this way, nanocomplexes combining chitosan and gold take advantages of both materials for biological applications [29–33], representing a promising material for DNA carriers development [5, 10, 12, 14].

There are a wide variety of biological methods to synthesize AuNPs, comprising the use of carbohydrates, microorganisms, enzymes, vitamins, and biopolymers, among others [34]. Between the so-called green synthesis [35], the use of the one-pot synthesis method, using chitosan as reducing and stabilizing agent, has proven to be more tunable and safe for biomedical applications [36, 37]. Same as traditional synthesis methods, in the case of one-pot synthesis, obtained particle size depends on the concentration of reducing agent; in this way, in the particular case of chitosan oligosaccharide, chitosan/gold ratio must be controlled in order to obtain different particle size, being the molecular weight of chitosan an important factor for nanoparticles’ size and shape distribution. This work was aimed at the evaluation of different chitosan-AuNP-based nanocomplexes for DNA transfection, including green/one-pot synthesis with 5 kDa chitosan oligosaccharide which is a lower molecular weight than the ones reported for similar applications [36, 38].

Transfection tests were performed in the HEK-293 cell line (*Homo sapiens* (human)-epithelial morphology-embryonic kidney-fetus) with pSV-β-Gal and pIRES2-EGFP plasmids. These tests were selected based on transfection efficiency and cytotoxicity which are cell type dependent and that, according to Corsi et. al. [4], transfection is more favorable for cell line HEK-293 in comparison with MG63 and mesenchymal stem cells.

## Methods

### Nanocomposite Synthesis

**Fig. 2:**
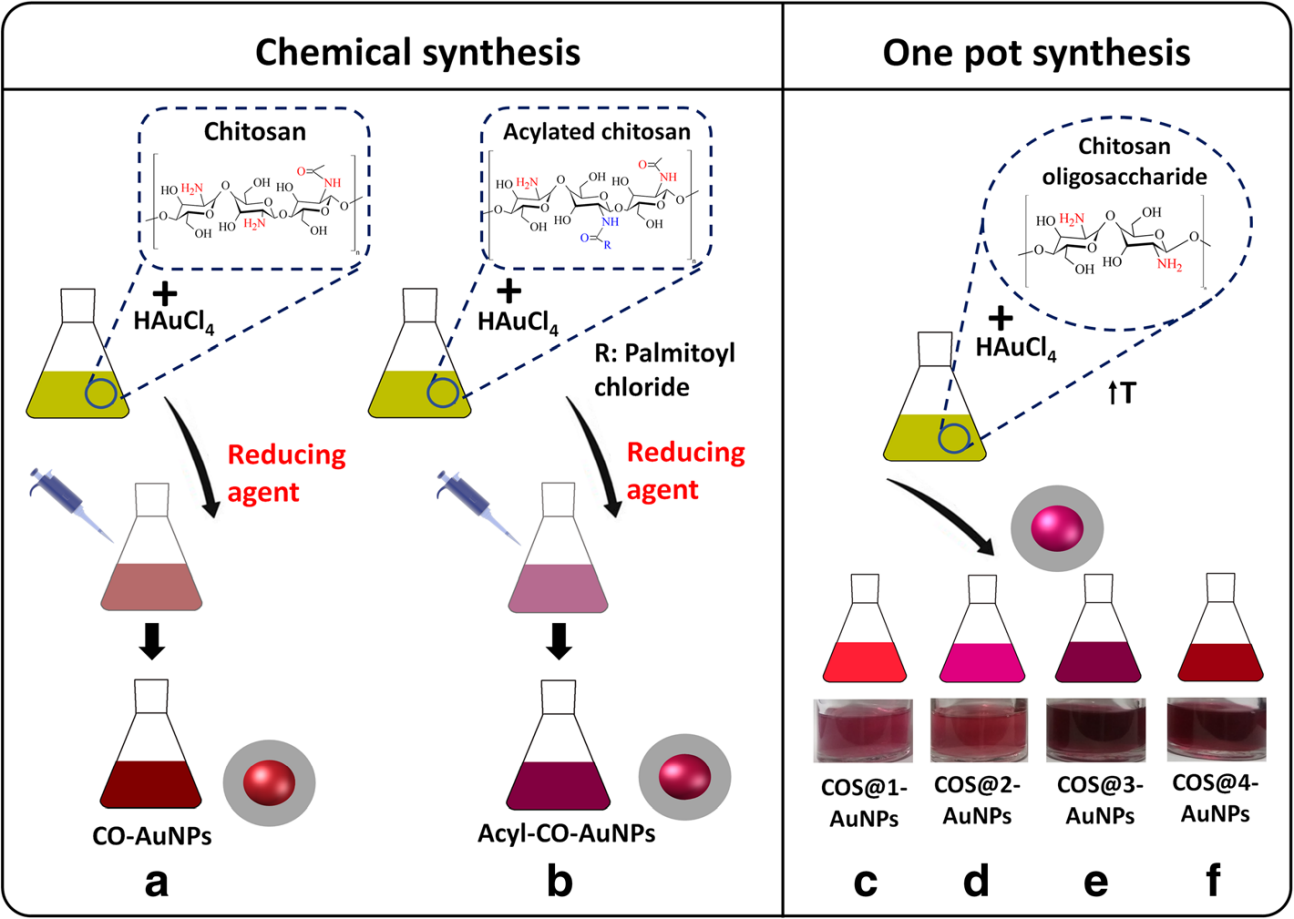
Schematic diagram for each synthesis: **a**–**b** chemical synthesis for gold nanoparticles with chitosan in its simple form (CO-AuNPs) and with hydrophobically modified acyl groups (Acyl-CO-AuNPs) and **c**–**f** green/one-pot synthesis for short chain chitosan (chitosan oligosaccharide, COS@n-AuNPs)

Each experiment was carried out with gold nanoparticles with modified and unmodified chitosan; two main synthesis methods were used: one of them involving chemical synthesis for gold nanoparticles with chitosan in its simple form (CO-AuNPs) and hydrophobically modified chitosan with acyl groups (Acyl-CO-AuNPs) and the other one a green/one-pot synthesis using short chain chitosan (chitosan oligosaccharide, COS@n-AuNPs). Figure 2 shows a schematic diagram for each synthesis; the corresponding experimental procedures are described below.

### Materials

Gold(III) chloride trihydrate (HAuCl_4_∙3H_2_O), sodium borohydride (NaBH_4_), low molecular weight chitosan (50-190 kDa, 75% deacetylation degree), and chitosan oligosaccharide (low molecular weight, 5 kDa) were obtained from Sigma Aldrich. All glassware were washed and left in a bath of freshly prepared aqua regia (HCl: HNO_3_, 3:1 *v*/*v*) for 24 h, and rinsed thoroughly with Mili-Q water before use, to remove any trace of metal [39].

### Chitosan-Gold Nanoparticles (CO-AuNPs)

Chitosan-gold nanoparticle synthesis was carried out by following Huang et. al. [9]’s methodology. For this, 20 mg of low molecular weight chitosan solution (2 mg/ml) was added to 10 ml of 1% acetic acid and mixed with vortex until complete dissolution and stored overnight. Two milliliters of the obtained solution was filtered using a 0.22 μm polyethersulfone syringe filter, then it was mixed with 1 ml of 10 mM HAuCl_4_∙3H_2_O solution by using strong stirring for 30 min. 0.4 ml of freshly prepared 100 mM NaBH_4_ cold solution was used as reducing agent; it was added by dropping while stirring, and rapid color turning from yellow to wine-red was observed; after that, stirring continued for a total time of 2 h (Fig. 2a).

### Acylated Chitosan-Gold Nanoparticles (Acyl-CO-AuNPs)

Caproyl chloride (C_6_H_11_ClO, Sigma Aldrich) was used for the synthesis of acylated chitosan-gold nanoparticles.

#### Gelation

Chitosan (low molecular weight) acylation was performed by following the method reported by Remant Bahadur et al. [6] with slight modifications. For this, 0.83 g of chitosan was added to 100 ml of 1% acetic acid solution under magnetic stirring which continued for 24 h. Five milliliters of this solution was filtered with a 0.22-μm polyethersulfone syringe filter, and then its pH was adjusted to 7.2 with a 0.1-M sodium hydroxide (NaOH) solution slowly added under vigorous stirring. One milliliter of caproyl chloride was added to 5 ml of this last solution and the resulted mixture was stirred for 5 h. The pH of the mixture was adjusted to 6.8–7 with a hydroxide solution. The obtained gel was precipitated with acetone and centrifuged at 5000 rpm for 20 min at 4 °C. Excess of caproic acid was removed by washing three times with methanol (50–60 °C) and decanting, letting it dry for 3 days on stove. This gel was used for the nanocomposite synthesis.

#### Nanocomposites

One milliliter of a 10-mM HAuCl_4_∙3H_2_O solution was added to 2 ml of gel diluted at 0.33 % in a 0.1 M HCl solution, stirring for 1 h. Finally, adding to this the last 0.4 ml of a 0.1-M NaBH_4_ freshly prepared cold solution while stirring, gold nanoparticle formation was evident with the pink/red color turning before completing 2 h of continuous stirring (Fig. 2b).

### Chitosan Oligosaccharide-Gold Nanoparticles (COS@n-AuNPs)

COS@n-AuNPs were prepared by following a modified methodology reported by Manivasagan et al. [20]. Chitosan oligosaccharide (COS) was added to 10 ml of HAuCl_4_∙3H_2_O solution and stirred for 60 min at 80 °C. Four syntheses were performed by varying the amount of COS (100 and 200 mg) and the concentration of gold in the HAuCl_4_∙3H_2_O solution (0.003 and 0.017 wt%). Table 1 shows the parameters used in AuNP synthesis for each experiment. Gold nanoparticle formation was evident with the wine-red/pink-red color turning, registering color change at different times on each case, depending on the amount of reducing agent and gold precursor. The obtained nanocomposites were purified by centrifuging at 12,000*g* for 30 min (Fig. 2c–f).

**Table 1:** Parameters used in AuNP synthesis for each experiment

Experiment	Average MW chitosan (Da)	Chitosan (wt%)	HAuCl_4_∙3H_2_O concentration (wt%)	Temperature (°C)	Time (min)
CO-AuNPs^a^	120000	0.12	0.100	25	150
Acyl-CO-AuNPs^b^	120000	0.69	0.100	25	90
COS@1-AuNPs^c^	5000	0.99	0.003	80	60
COS@2-AuNPs^d^	5000	1.96	0.003	80	60
COS@3-AuNPs^e^	5000	0.99	0.017	80	60
COS@4-AuNPs^f^	5000	1.96	0.017	80	60

^a^Chitosan

^b^Acyl Chitosan

^c–f^Chitosan oligosaccharide

### Nanocomposite Characterization

#### Infrared Spectroscopy

Chitosan used for CO-AuNPs, acylated chitosan for Acyl-CO-AuNPs, and chitosan oligosaccharide for series of COS@n-AuNPs were characterized by FTIR spectrometry (Spectrum One, Perkin Elmer) to confirm acylation of Acyl-CO polymer by comparing the intensity of characteristic bands from functional groups between samples. Substitution degree (SD) was estimated from IR spectra information, according to Remant Bahadur et. al. [6].

#### ξ-Potential

Zetasizer (Nano Z, Malvern) was used to evaluate the composite surface potential. Positive potential is expected for nanocomposites for successful adhesion to DNA to form complexes. **ξ-**Potential was evaluated before and after complex formation to confirm changes after DNA adhesion. 1.5 ml of appropriate diluted samples were used for surface potential measurements, using a capillary cell (DTS 1060, Malvern).

#### UV-Vis Spectroscopy

UV-Vis spectra (UV-1800 m, Shimadzu), in a range of 400 to 700 nm, were obtained for preliminary confirmation of gold nanoparticle formation by means of the surface plasmon resonance band around 530 nm. Complementary, the spectra from freshly prepared nanocomposites were used to qualitatively evaluate photostability of samples; this was done by comparing with spectra obtained from the same samples 150 days after synthesis, by following changes around surface plasmon resonance band, choosing one sample for each method, and using COS@2-AuNPs as the most representative for COS@n-AuNP series. Quartz cell was used as the sample’s container and deionized water as blank.

#### Transmission Electron Microscopy (TEM)

TEM microscopy (JEM 1010, JEOL) was used for determination of shape and particle size distribution. For TEM images, nanocomposite samples were prepared by depositing 10 μl of solution over 200 mesh copper grid support, covered with formvar, and drying at room temperature for 15 min. Three images for each sample were selected and analyzed by means of a home-made imaging processing program developed under Matlab® software.

### Complex Formation and Adhesion Degree

#### Nanocomposite-pDNA

pSV-β-Gal (6.82 kb) and pIRES2-EGFP (5.3 kb) (pDNA) were isolated from transformed DH5α *Escherichia coli*. Plasmid purification was performed by alkaline lysis, using Mo Bio® UltraClean Microbial DNA Isolation Kit. Agarose gel electrophoresis was used to verify the structural integrity of plasmids, using a high-performance ultraviolet transilluminator, acquiring images with a Kodak Gel Logic 100 Digital Imaging System. Plasmid DNA concentration and purity was validated with Synergy™ 2 Multi-Detection Microplate Reader using Gen5 program. Complexes were obtained by mixing plasmid DNA (pDNA) with each type of gold-chitosan nanoparticle nanocomposites in no serum Dulbecco’s modified Eagle’s medium (DMEM, Sigma-Aldrich) at plasmid quantities between 200 and 400 ng. pDNA adhesion to nanocomposite was evaluated by electrophoresis using agarose gel at 0.8% and voltage of 90 V for 60 min.

### Chitosan-Gold Nanoparticle-pDNA Complex Transfection into HEK-293 Cells

#### Cell Culture

HEK-293 *Homo sapiens* (human)-epithelial morphology-embryonic kidney-fetus cells were cultivated in Dulbecco’s modified Eagle’s medium with fetal bovine serum (SFB, Sigma-Aldrich) at 37 °C under 5% CO_2_-containing atmosphere. For pDNA transfection, HEK-293 cells were seeded at 12,000 cells per well in a 96-well plate and incubated for 24 h at 37 °C in 5% CO_2_-containing atmosphere, obtaining 90% confluence.

#### Transfection Efficiency with pIRES2-EGFP

In vitro pDNA nanocomposite functionalization was performed by pouring culture medium from wells until barely covering cells and adding the complex solution on each well (15 μl of nanocomposites and 200 ng of DNA), incubating for 2 h at the same culture conditions. After that, 500 μl of complete DMEM was added for incubation for 48 h. pIRES2-EGFP transfection was directly evaluated by fluorescence microscopy (Axio Vert.A1, Carl Zeiss).

#### Transfection Efficiency with pSV-β-Gal

X-gal histochemistry was used to evaluate the β-galactosidase activity, by modifying pSV-β-Gal plasmid expression on HEK-293 cell line; in this methodology, the transfected cells turned blue. In vitro pDNA nanocomposite functionalization was performed as explained above, incubating for 2 h at the same culture conditions. After that, 500 μl of complete DMEM was added for incubation for 24 h, changing medium and incubating for an additional 24 h. Wells were washed two times with 50 μl sterile phosphate-buffered saline (PBS) 1× after removing culture medium, then cells were fixed with 2% formaldehyde and 0.2% Glutaraldehyde mixture for 5 min; after that, they were washed two times with 50 μl PBS 1×. Fixer was removed before washing two times with 50 μl PBS 1× and then with a mixture of 0.4 mg/ml X-gal (5-bromo-4-chloro-3-indolyl-β-D-galactopyranoside, Sigma-Aldrich) diluted in 5 mM potassium ferrocyanide (Meyer) and magnesium chloride 2 mM (Meyer) on PBS 1×. Then, the cells were incubated for 24 h at room temperature. β-galactosidase expression was observed by brightfield microscopy (AE2000, Motic), taking 5 fields per well with × 40 objective, expecting the transfected cells to turn blue. Transfection efficiency was evaluated by comparing cells with β-galactosidase expression (turned blue) against total cells per field. LipofectAMINE^TM^2000 (30 μl) was used as a positive control for every test.

### Cell Viability by Dye Exclusion Method

Microcultures were used 24 h after cell passage. Culture medium was removed, and cells were washed with PBS 1× before adding 40 μl of complex solution. After that, cells were incubated for 2 h at 37 ° C at 5% CO_2_-containing atmosphere. For dye exclusion, the complex solution was washed from cells with 50 μl PBS 1× and 20 μl Trypan blue (0.2 % on PBS 1×) was incorporated. With this last mixture, cells were incubated for 10 min at 37 °C and 5% CO_2_-containing atmosphere. After this last incubation, the dye was removed from wells and cleaned by washing for two times with 50 μl PBS 1×. Cells were observed by brightfield microscopy, counting blue dyed cells (dead or damaged) against total cells per field. Cell viability tests were obtained for different complex concentrations on water, from 0.1 mM to 3 mM, CO-AuNPs and Acyl-CO, at 3 mM; COS@3-AuNPs and COS@4-AuNPs, at 0.5 mM; and the lowest concentration (0.1 mM) for COS@1-AuNPs and COS@2-AuNPs.

Software *Image J* (*Open Source Software* (*OSS*) *license*) together with the plugin *Image-based Tool for Counting Nuclei-ICTN* were used to count cells for transfection and viability tests.

## Results and Discussions

### Nanocomposite Synthesis and Characterization

Among the three kinds of complexes synthesized in this work, COS@n-AuNP deserves special attention since they were synthesized without the use of toxic reagents, like sodium borohydride or cetyltrimethylammonium bromide (CTAB). It has been reported that chitosan is suitable as a reducing and stabilizing agent and optimal concentration for synthesizing AuNP complexes at desired sizes and shapes depending on multiple factors: reaction time, temperature, and degree of chitosan deacetylation and its molecular weight, among others [38, 40]. Under this consideration, chitosan molecular weight was kept constant at 5 kDa for the four COS@n-AuNP syntheses, taking HAuCl_4_∙3H_2_O and chitosan solutions at different concentrations.

**Fig. 3:**
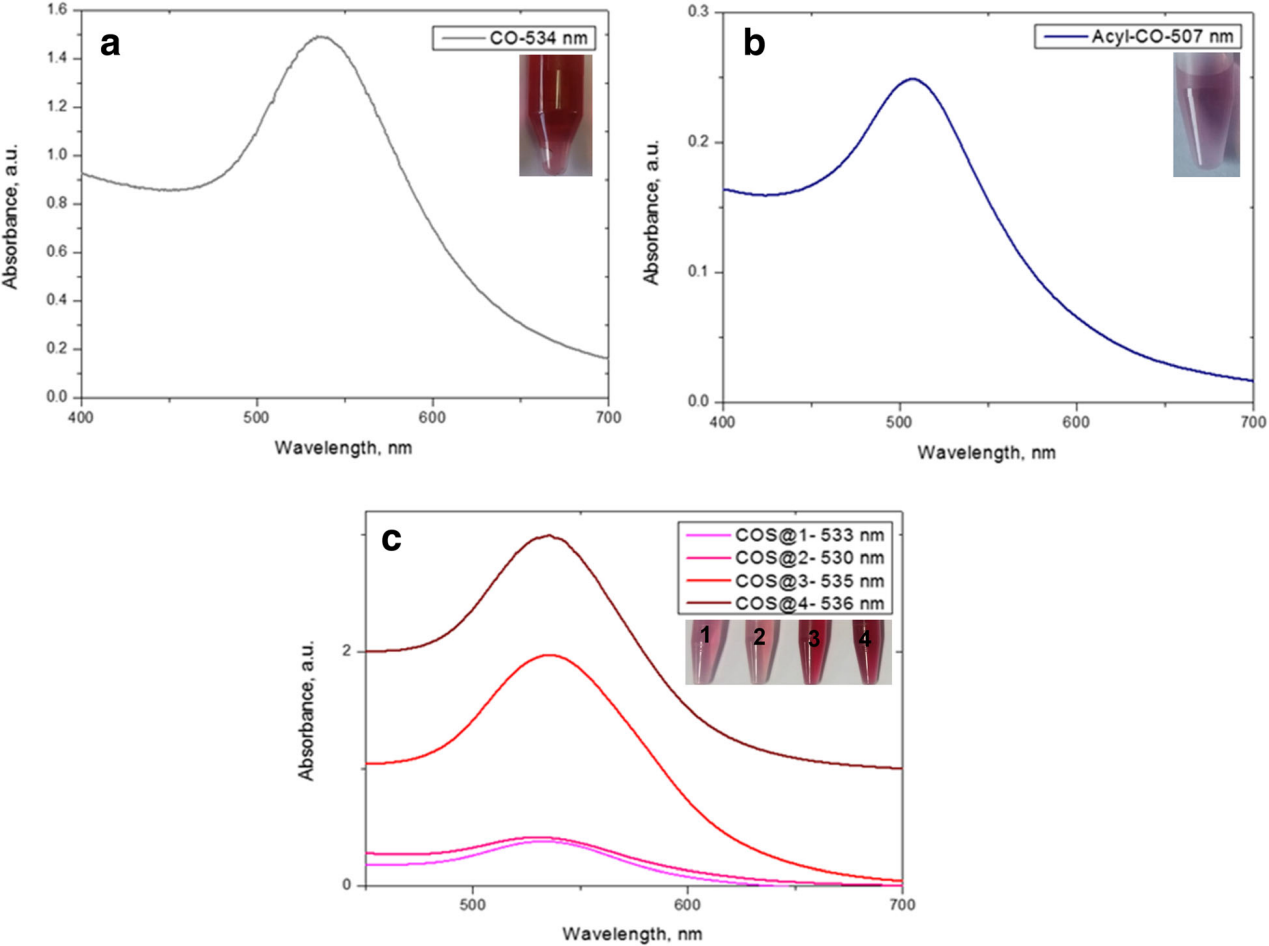
UV-Vis spectra for nanocomposites. Values in the insets correspond to the maximum wavelength of the localized surface plasmon resonance. These maxima were as follows: for CO-AuNPs at 534 nm (0.1 M NaBH_4_) (**a**), for Acyl-CO-AuNPs at 507 nm (**b**), and for COS-@n-AuNP chitosan oligosaccharide at 533, 530, 535, 536 nm for each at 1–4 different thickness nanocomposite preparations (**c**)

Figure 3 shows UV-Vis absorption spectra from AuNPs-chitosan nanocomposites synthesized in this work. Plasmon bands centered at 534 nm for chitosan (CO-AuNPs), 507 nm for acylated chitosan (Acyl-CO-AuNPs), and for the various chitosan oligosaccharide at 533 nm (COS@1-AuNPs), 530 nm (COS@2-AuNPs), 535 nm (COS@3-AuNPs), and 536 nm (COS@4-AuNPs) confirm the presence of gold nanoparticles. Insets in this figure show pictures of the obtained nanocomposites which coloration depends on the nanoparticles’ concentration, size, shape, and surface functionalization.

#### Stability of Nanocomposites

**Fig. 4:**
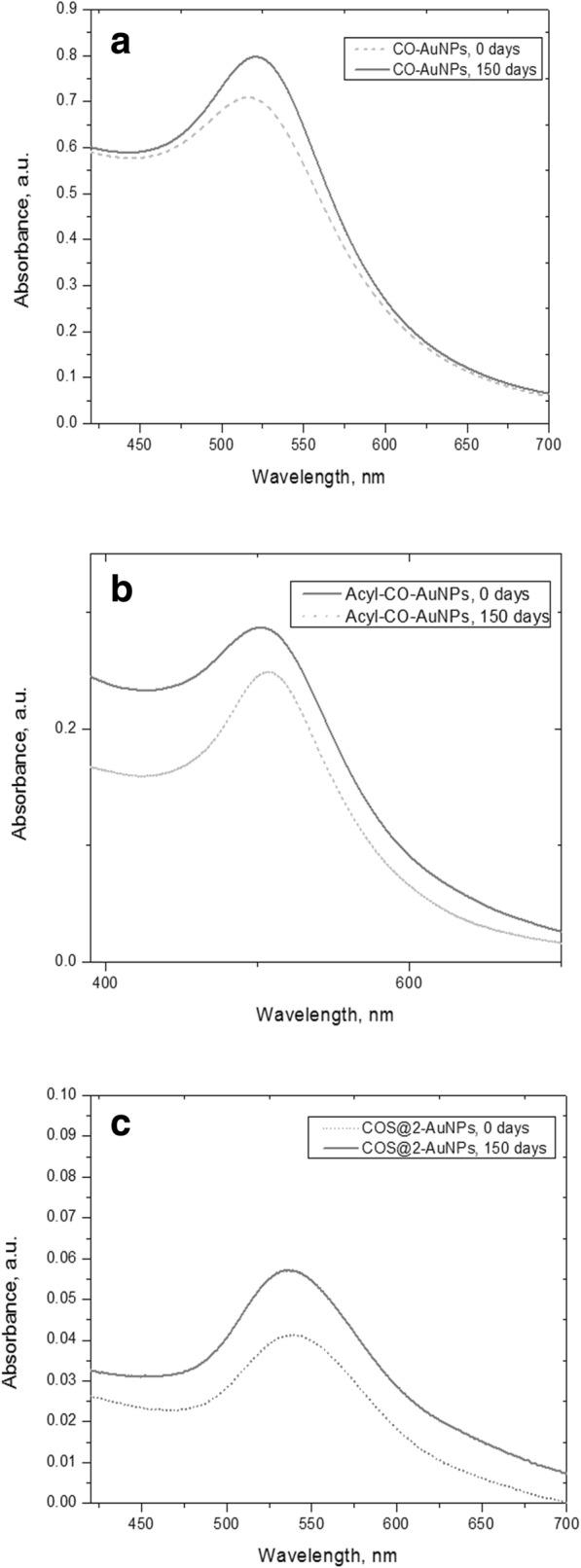
Stability of gold nanoparticle nanocomposites by UV-Vis absorption spectra obtained 150 days after synthesis: chemically synthesized CO-AuNPs (**a**) and Acyl-CO-AuNPs (**b**) and green/one-pot synthesis for COS@2-AuNPs (**c**)

Figure 4 shows the comparison between UV-Vis absorption spectra from freshly synthesized nanocomposites and 150 days after synthesis; as can be observed, the two spectra for each case remain essentially the same, no significant shifting of the maximum of resonance bands, and no evidence of additional peaks. These results qualitatively suggest high stability of the obtained nanocomposites.

**Fig. 5:**
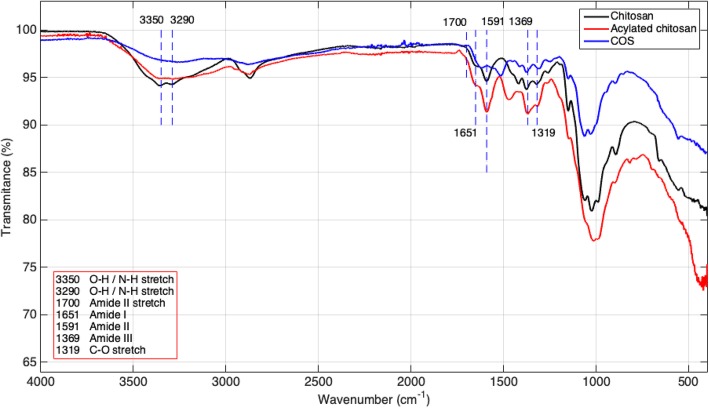
FT-IR spectra of chitosan, acyl-chitosan, and chitosan oligosaccharide. Band at 1591 cm^−1^ corresponds to secondary amides; 3200–3500 cm^−11^ regions correspond to N-H and O-H vibrations

Infrared spectroscopies for the three different chitosan samples are shown in Fig. 5. This technique was used for the identification of the acylated chitosan by comparing its absorption bands inside the infrared (IR) region (which are characteristics of the chemical structure) with the IR database. Some characteristic IR bands are worth to point out: chitosan (50–190 kDa, 75% deacetylation degree) bands at 1656 cm^−1^, 1593 cm^−1^, and 1373 cm^−1^ correspond to amide groups (amides I, II, and III, respectively); the band at 2895 cm^−1^ corresponds to C-H bonds, and the ones between 3200–3500 cm^−1^ to N-H and O-H bonds. For acylated chitosan, IR bands were obtained at 1651 cm^−1^, 1591 cm^−1^, and 1369 cm^−1^ correspond to amide groups (amides I, II, and III, respectively), and the ones inside 3200–3500 cm^−1^ correspond to N-H and O-H bonds. Chitosan acylation was confirmed according to IR spectroscopy data [7]: the band at 1591 cm^−1^ was lower for acylated chitosan compared to native chitosan due to a larger number of secondary amides on acylated chitosan. Bands inside 3200–3500 cm^−1^, characteristic to N-H and O-H vibrations in native chitosan, disappears for acylated chitosan and chitosan oligosaccharide due to primary amide reduction.

The degree of substitution for acylated chitosan was estimated, according to Bahadur et. al. [6], by the following expression:

$$ \% DS=\left(\left(\frac{A_{1651}}{A_{3350}}\right)-0.25\right)\times 100=75.16781\% $$

where *A*_1651_ = 1.973965 and *A*_3350_ = 1.977278 correspond to absorbance (for amide I and OH vibration, respectively) of acylated chitosan at 1631 cm^−1^ and 3350 cm^−1^, respectively, and the 0.25 corresponds to the groups of free amines. A degree of N-acylation of 75% was obtained.

#### ξ Potential

ξ-Potential values were obtained for each composite as follows (Table 2, column “ξ-Potential”): 43.3 mV (CO-AuNPs), 40.2 mV (Acyl-CO-AuNPs), 52.2 mV (COS@1-AuNPs), 55.3 mV (COS@2-AuNPs), 51.6 mV (COS@3-AuNPs), and 46.3 mV (COS@4-AuNPs). Potential for all nanocomposites was positive due to the amine groups from chitosan as it was planned in order to achieve DNA complex formation by electrostatic forces. After complex formation, reduction of ξ-potential values confirmed the successful adhesion of DNA (Table 2, column “ξ-Potential/DNA”).

**Table 2:** Physical parameters summary

Experiment	UV-Vis λmax (nm)	ξ-Potential	ξ-Potential/DNA	Average TEM diameter (nm)
CO^a^	534	43.3	36.9	3.492 ± 1.343
Acyl-CO^b^	507	40.2	34.4	4.659 ± 1.975
COS@1^c^	533	52.2	44.9	7.385 ± 2.427
COS@2^d^	530	55.3	46.9	15.642 ± 3.541
COS@3^e^	535	51.6	44.9	10.061 ± 4.727
COS@4^f^	536	46.3	31.6	13.992 ± 3.984

^a^Chitosan

^b^Acyl chitosan

^c–f^Chitosan oligosaccharide

#### Transmission Electron Microscopy (TEM)

**Fig. 6:**
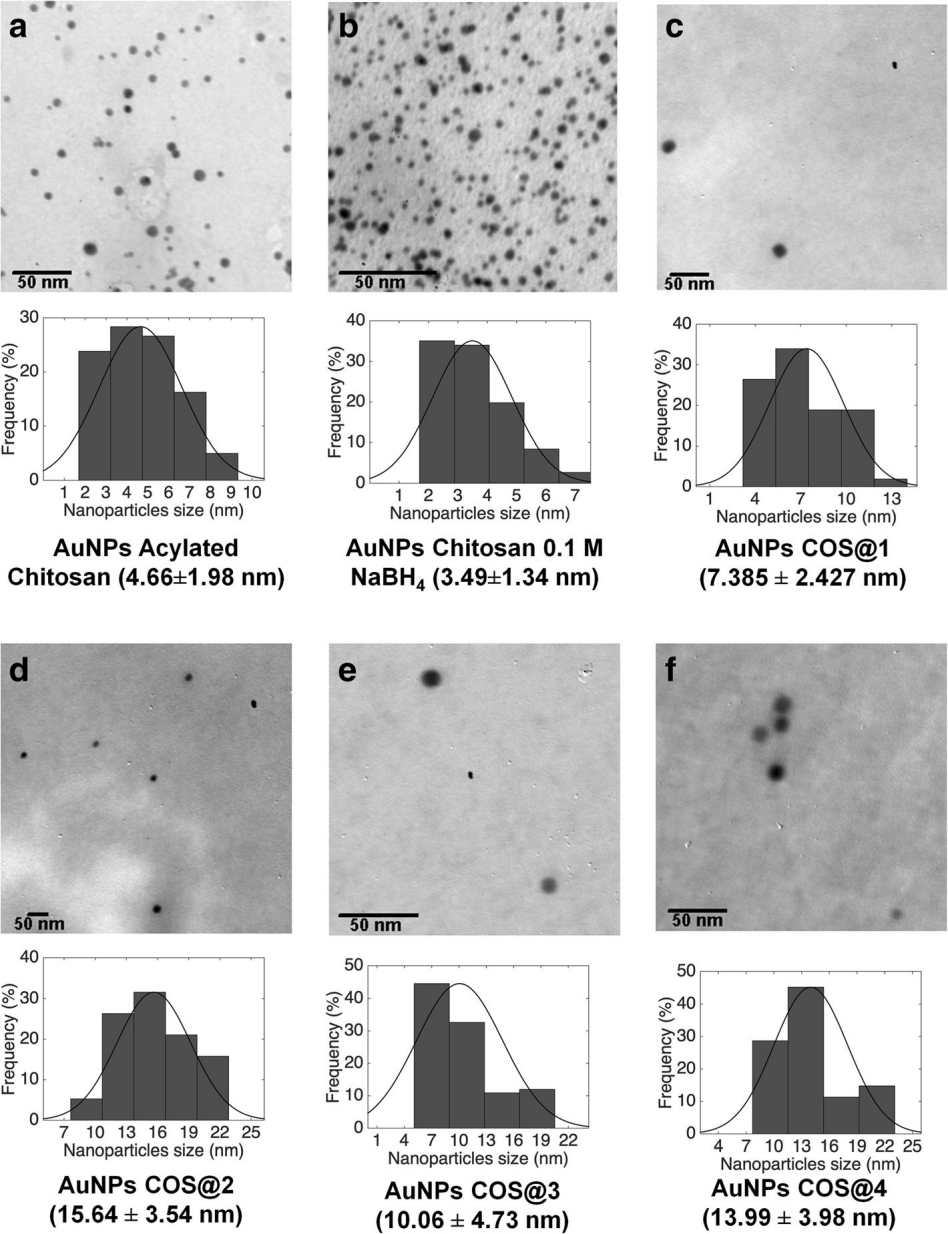
TEM micrographs from **a** 4.7 nm acylated chitosan AuNPs, **b** 3.5 nm chitosan AuNPs (0.1 M NaBH_4_), **c** 7.4 nm oligosaccharide chitosan AuNPs (COS@1), **d** 15.6 nm oligosaccharide chitosan AuNPs (COS@2), **e** 10 nm oligosaccharide chitosan AuNPs (COS@3), and **f** 14 nm oligosaccharide chitosan AuNPs (COS@4)

Figure 6 shows the representative TEM micrographs for each nanocomposite, along with nanoparticle size histograms for size distribution calculation. Size distributions for each nanocomposite were obtained as follows: three TEM images for each sample were obtained and analyzed with a home-made image processing software developed with Matlab® and the nanoparticle count was 500 for Acyl-CO-AuNPs, 1000 for CO-AuNPs, and 100 for COS@n-AuNP series. The average size for each sample is summarized in Table 2, column “Average TEM diameter (nm).” It is worth to point out that AuNPs synthesized with chitosan oligosaccharide resulted spherical, with a better size distribution as compared with similar ones obtained with other green synthesis methodologies reported in the literature [36, 38, 41–43].

#### Complex Formation and Adhesion Degree: Nanocomposite-pDNA

**Fig. 7:**
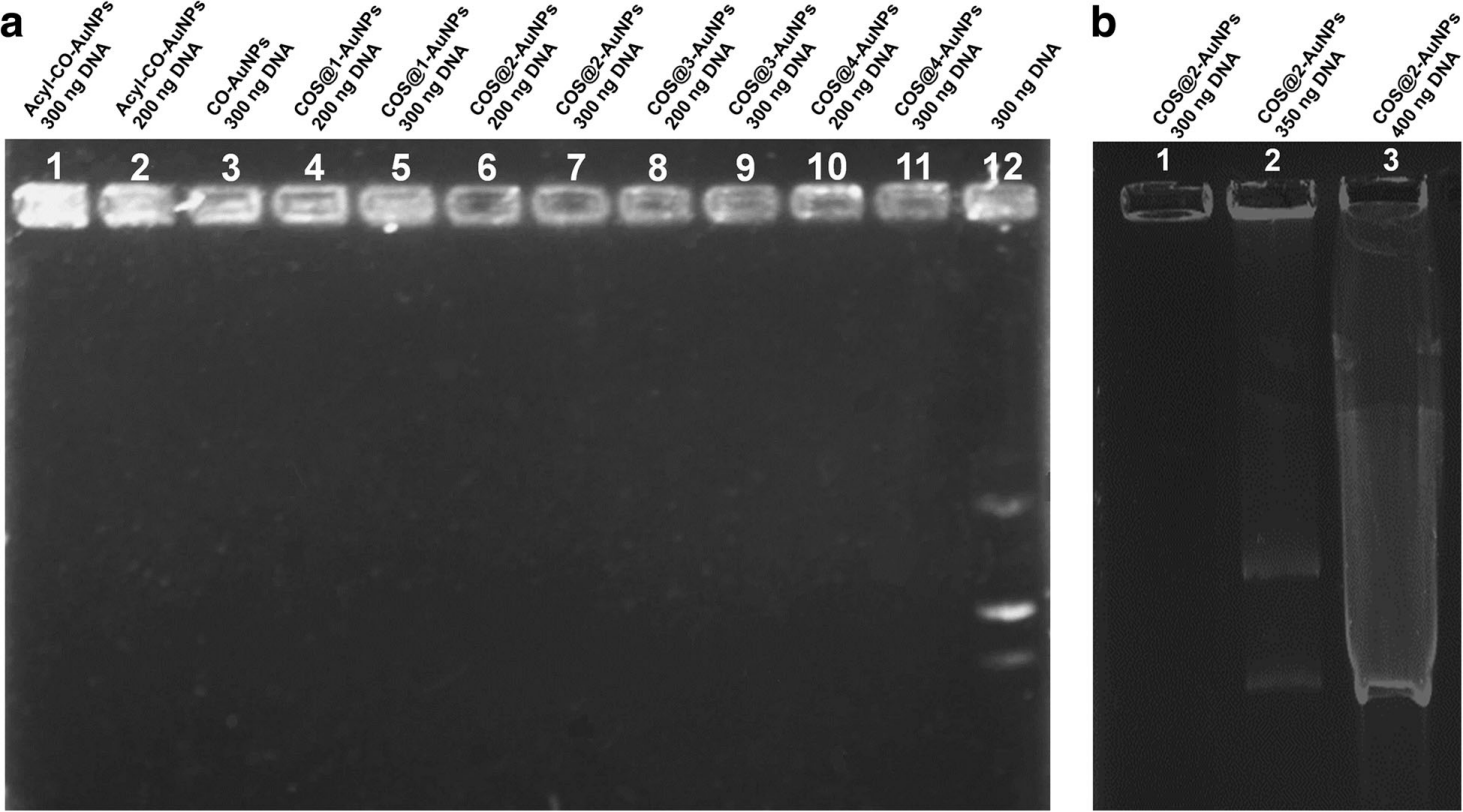
**a** Electropherograms for nanocomplexes with 200 and 300 ng pDNA concentrations. Lane 12 corresponds to pure pDNA. **b** CO-AuNPs and Acyl-CO-AuNPs with 300 ng of pDNA, pure pDNA at lane 3. **c** COS@2-AuNPs nanocomplex with 300 and 350 ng of DNA, 400 ng of pure pDNA at lane 3. **d** same CO-AuNPs and Acyl-CO-AuNPs at 1:10 dilution, with respectively pure DNA control at lane 3

Electropherograms of all pDNA complexes and pure DNA were carried out, taking different concentrations of the plasmid; results are shown in Fig. 7. Figure 7a shows the corresponding electropherograms for the pure plasmid and all complexes carried with 200 and 300 ng of the plasmid. Distribution of nanocomposite samples for these electropherogram goes as follows: Acyl-CO-AuNPs (300 and 200 ng) at lanes 1 and 2, CO-AuNPs (300 ng) at lane 3, COS@1-AuNPs (300 and 200 ng) at 4 and 5, COS@2-AuNPs (300 and 200 ng) at 6 and 7, COS@3-AuNPs (300 and 200 ng) at 8 and 9, COS@4-AuNPs (300 and 200 ng) at 10 and 11, and 300 ng of pure pDNA at lane 12. For all tests, 10 μl of complex solution was used. This figure shows that only the pDNA travels in the gel (as evident in lane 12) and the other pDNA complexes remain in their corresponding wells, confirming by this way the pDNA adhesion on all complexes. This is due to the interaction of the positive charges of the amino groups from chitosan, which remains on the gold nanoparticle surface, and the negative charges of the phosphate groups of DNA helixes.

To evaluate the retention ability of COS@2 complexes, eletropherograms for the corresponding pDNA complex were carried out with the same COS@2 concentration but different pDNA concentrations (300 ng, 350 ng, and 400 ng); the results are shown in Fig. 7b. Lanes 1, 2, and 3 on this figure correspond to plasmid concentration of 300 ng, 350 ng, and 400 ng, respectively; results in lane 1 is in perfect agreement with the corresponding result in Fig. 7a (lane 7) and, both results in lane 2 and 3 of Fig. 7b, indicate that retention power of this complex is below 350 ng of the plasmid.

### Chitosan-Gold Nanoparticle-pDNA Complex Transfection into HEK-293 Cells

**Fig. 8:**
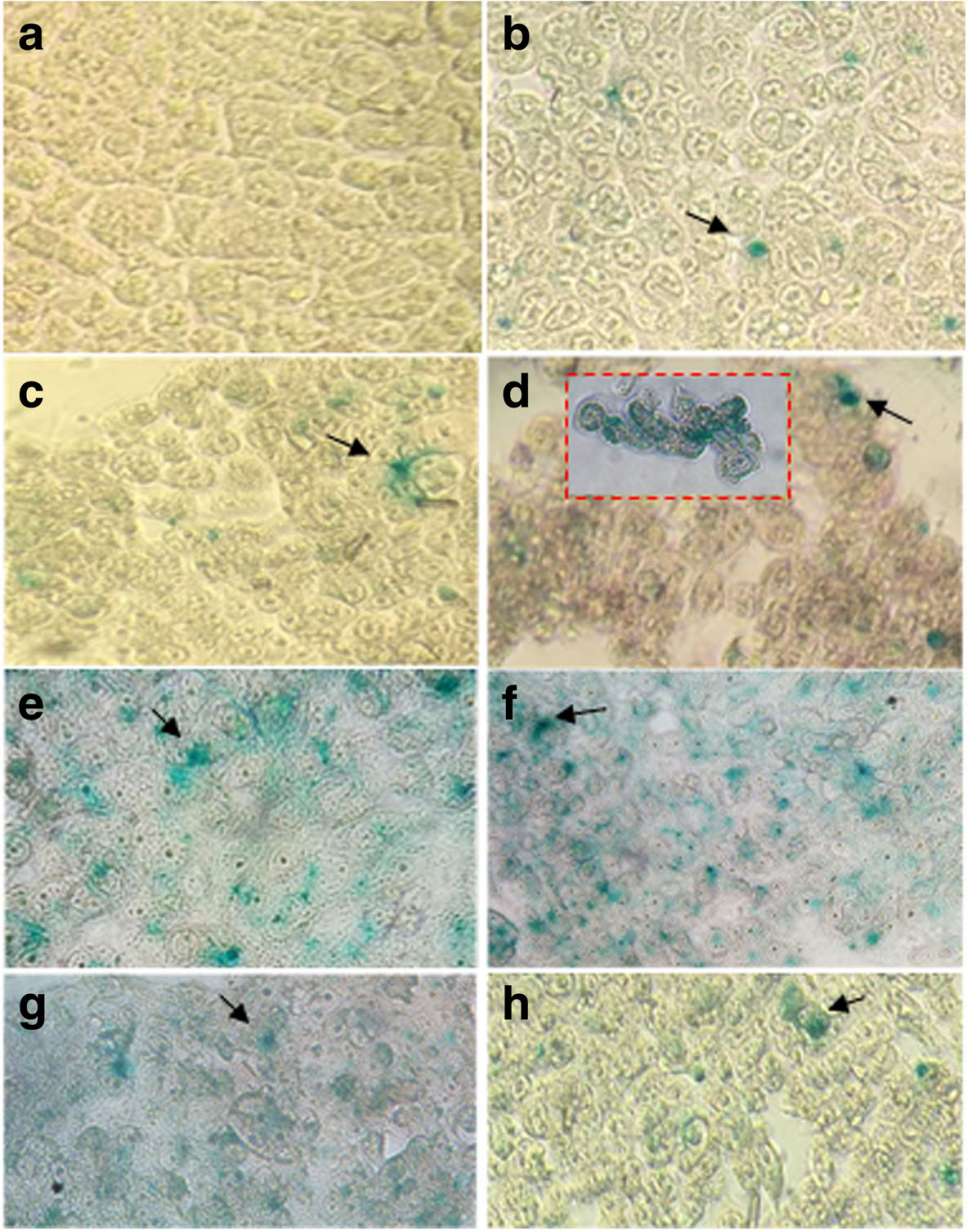
HEK-293 *Homo sapiens* (human)-epithelial morphology-embryonic kidney-fetus cell transfection tests (pSV-β-Gal). **a** HEK-293 cells, **b** positive control: LipofectAMINE^TM^2000, **c** CO-AuNP, **d** Acyl-CO-AuNPs, **e** COS@1-AuNPs, **f** COS@2-AuNPs, **g** COS@3-AuNPs, and **h** COS@4-AuNPs. Arrows point to transfected cells

**Fig. 9:**
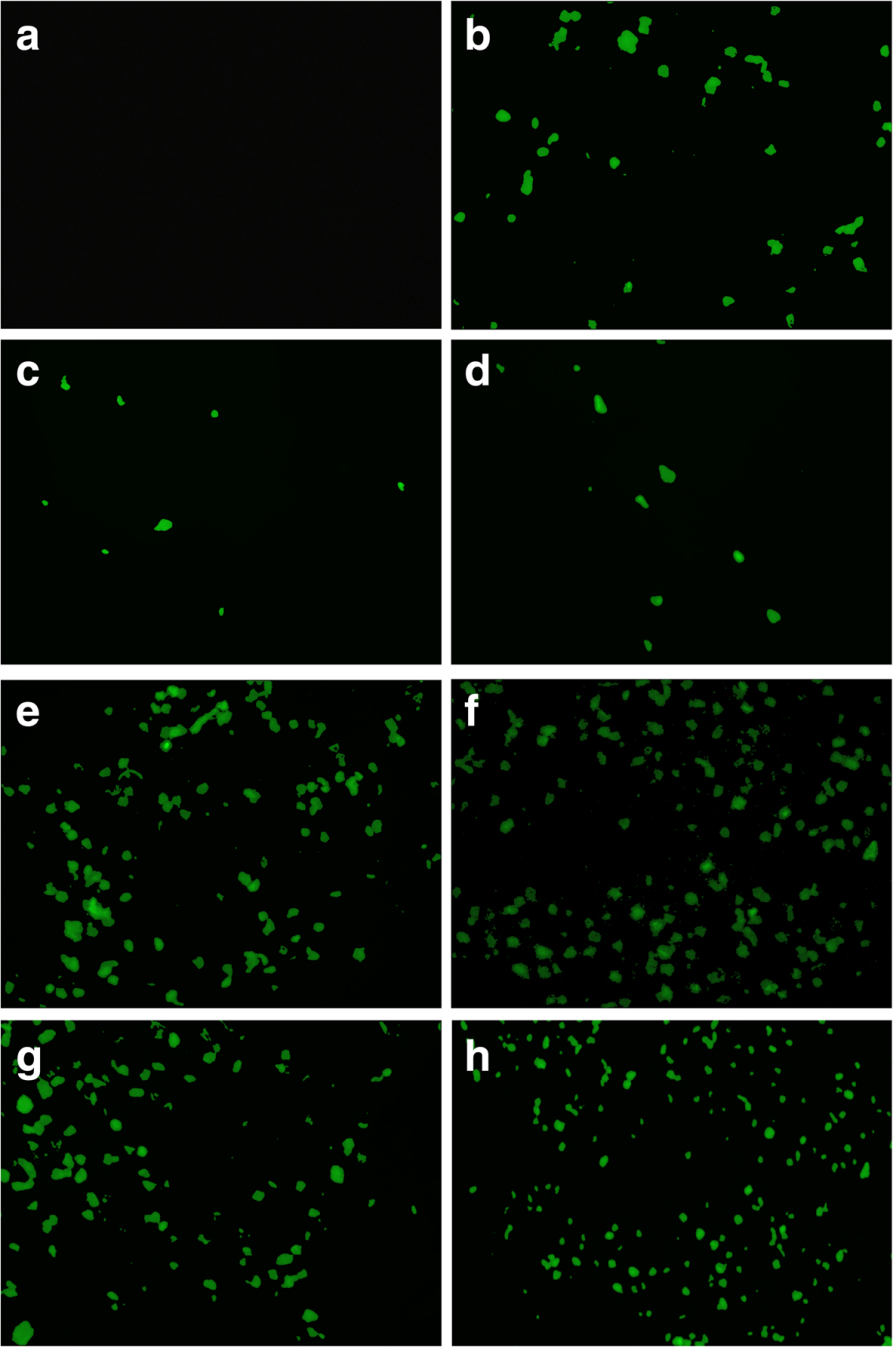
HEK-293 *Homo sapiens* (human)-epithelial morphology-embryonic kidney-fetus cell transfection tests (pIRES2-EGFP).**a** HEK-293 cells, **b** positive control: LipofectAMINE^TM^2000, **c** CO-AuNP, **d** Acyl-CO-AuNPs, **e** COS@1-AuNPs, **f** COS@2-AuNPs, **g** COS@3-AuNPs, and **h** COS@4-AuNPs. Arrows point to transfected cells

**Fig. 10:**
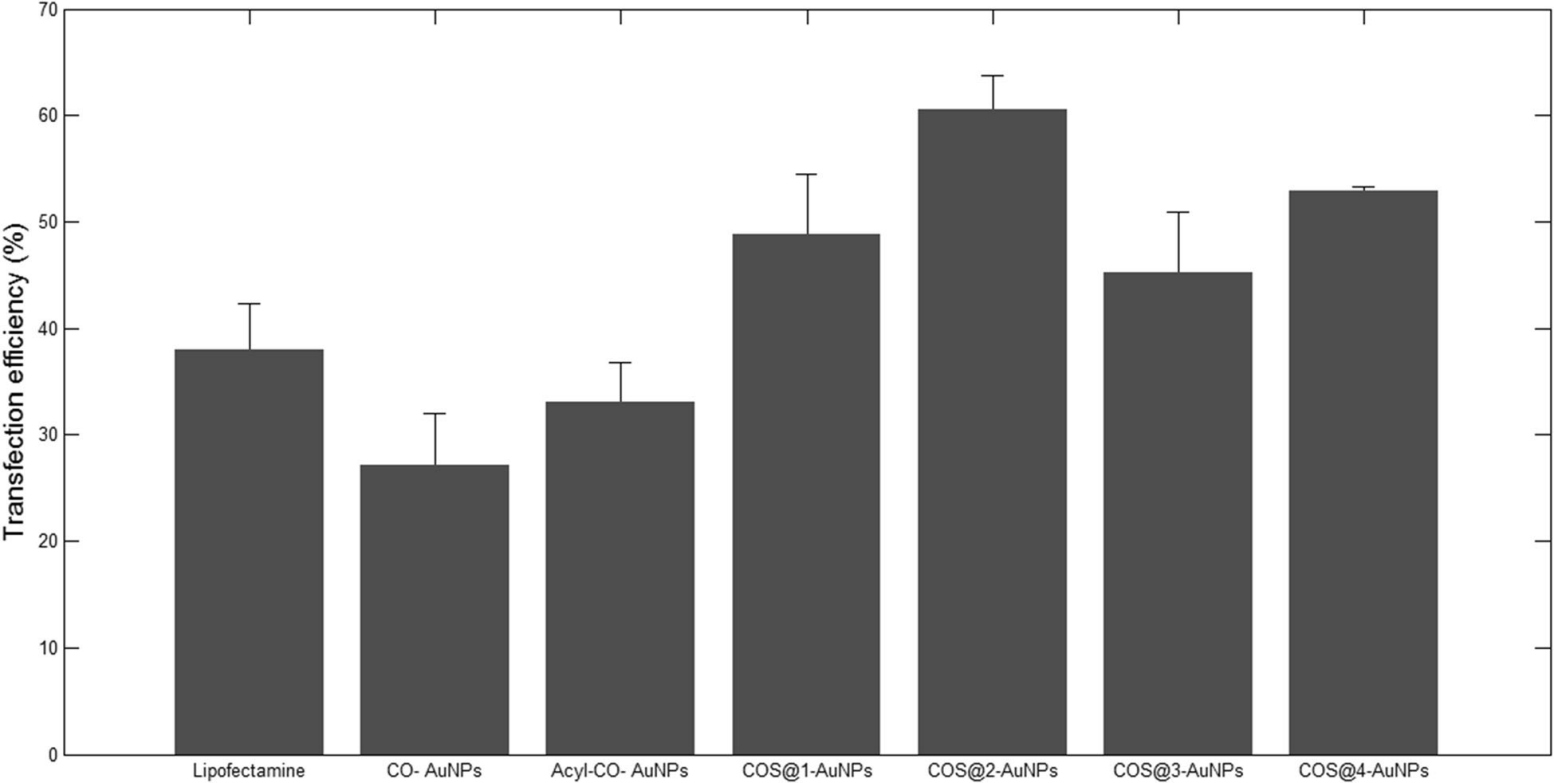
Transfection efficiency percentages obtained with the different nanocomplexes (pSV-β-Gal and pIRES2-EGFP test). HEK-293-transfected cells

As it was pointed out before, two different tests were made in order to evaluate pDNA transfection, taking advantage of the X-galactosidase activity of the pSV-β-Gal plasmid and the fluorescence activity for the case of the piRES2-EGFP plasmid. Figure 8 shows the micrographs of transfection test in HEK-293 cells by X-galactosidase activity on pSV-β-Gal; blue cells in this figure (pointed out by arrows) are some of the transfected cells, according to the corresponding methodology. On the other hand, Fig. 9 shows the fluorescence micrographs of transfected HEK-293 cells; this technique was used to evaluate transfection efficiency with pIRES2-EGFP plasmid. For both figures, (a) shows pure cells and (b) shows cells using LipofectAMINE^TM^2000 as a positive control. Corresponding transfections rates with each conjugate are shown as follows: (c) CO-AuNP, (d) Acyl-CO-AuNPs, (e) COS@1-AuNPs, (f) COS@2-AuNPs, (g) COS@3-AuNPs, and (h) COS@4-AuNPs. Transfection efficiency percentages obtained with the different complexes were as follows: CO-AuNP 27%, Acyl-CO-AuNP 33%, COS@1-AuNP 48%, COS@2-AuNP 60%, COS@3-AuNP 45%, and COS@4-AuNP 52%. Figure 10 shows the histograms for the transfection percentages obtained with each complex. Transfection efficiency was evaluated by comparing cells presenting pDNA expression against total cells per well. The highest transfection efficiencies were obtained with conjugates from COS@2-AuNP complexes; this can be explained by the highest chitosan/gold ratios used for this green method of synthesis. These results may complement those reported by Köping-Höggård et. al. [40], which suggests that the use of DNA conjugates with chitosan oligomers of different length affects the stability and transfection efficiency. The present work deserves additional consideration since the transfection efficiencies were improved in this case using low molecular weight chitosan oligosaccharides complexed with gold nanoparticles.

#### Dye Exclusion Test

**Fig. 11:**
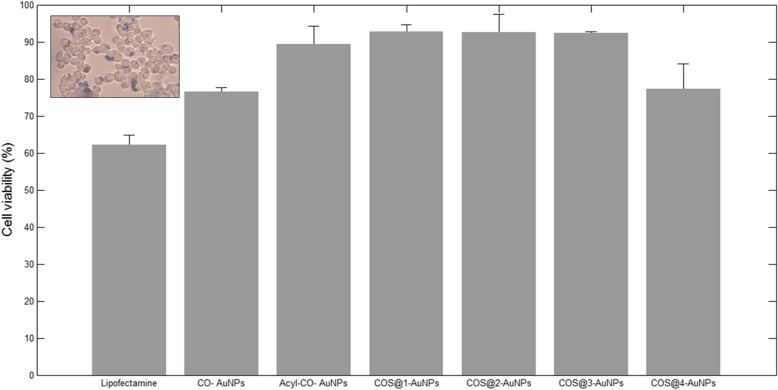
Cell viability percentages obtained with the different nanocomplexes (pSV-β-Gal and pIRES2-EGFP test). HEK-293 cells

Figure 11 (inset) shows the micrographs for cell viability evaluation. Blue dyed cells (dead or damaged) were compared against total cells per field*.* The highest cell viability was obtained with COS@1-AuNPs and COS@2-AuNPs, with 93.53% and 93.46%, respectively. It is interesting to note that COS@2-AuNPs also showed the best transfection efficiency. Among COS@n-AuNP series, COS@4-AuNPs showed lower viability (78.23%). As expected, tests with LipofectAMINE^TM^2000 and CO-AuNPs presented the lowest viability (61.45% and 77.85% respectively). Finally, viabilities with Acyl-CO and COS@3-AuNPs were 90.75% and 92.08 % respectively.

## Conclusions

Chitosan, acylated chitosan, and chitosan oligosaccharide nanocomposites with gold nanoparticles, featuring positive charges, were synthesized. They presented good stability in colloidal solution; this confirms the viable use of chitosan as a stabilizer and chitosan oligosaccharide as both reducing agent and stabilizer, and positive charges by its amino groups improved affinity with plasmid DNA. Size distributions of the synthesized gold nanoparticles were in a range of 3 to 15 nm. Gel electrophoresis and ξ-potential studies showed that plasmid DNA was successfully incorporated to the nanoparticles by interaction with the positive charges from chitosan at the surface of the nanocomposites. The best transfection efficiency, with 93.46% viability, was obtained with the COS@2-AuNP complexes, possibly due to its relatively high chitosan/gold ratio (1.96/0.003); this means larger effective surface for the anchoring of the plasmids by means of the electrostatic interaction of its phosphate groups with the chitosan amine functional groups. The COS@n-AuNP complexes obtained by the green/one-pot synthesis described in this work showed high transfection efficiency, as compared with those obtained with traditional chemical synthesis (CO-AuNPs and Acyl-CO-AuNPs), and can be proposed as promising green route-synthesized pDNA vehicles without the inconvenience of using toxic chemical reagents. Moreover, chitosan oligosaccharide as a reducing agent can be considered as a green synthesis method for AuNPs and renders spherical nanoparticles with good size distribution. This kind of safe and non-toxic routes of synthesis for nanocomplexes, like the ones proposed in this work, deserve further research in the search of obtaining more safe plasmid carriers for gene therapy applications.

## Data Availability

All datasets on which the conclusions of the manuscript rely are presented in the main paper.

## Abbreviations

Acyl-CO: Acylated chitosan
AuNPs: Gold nanoparticles
CO: Chitosan
COS: Chitosan oligosaccharide
DMEM: Dulbecco’s modified Eagle’s medium
FTIR: Fourier-transform infrared
PBS: Phosphate-buffered saline
pDNA: Plasmid Deoxyribonucleic acid
TEM: Transmission electron microscopy
